# Preferential Lineage-Specific Differentiation of Osteoblast-Derived Induced Pluripotent Stem Cells into Osteoprogenitors

**DOI:** 10.1155/2017/1513281

**Published:** 2017-01-30

**Authors:** Casey L. Roberts, Silvia S. Chen, Angela C. Murchison, Rebecca A. Ogle, Michael P. Francis, Roy C. Ogle, Patrick C. Sachs

**Affiliations:** ^1^Eastern Virginia Medical School, W. Olney Road, Norfolk, VA, USA; ^2^Institute of Regenerative Medicine, LifeNet Health, Concert Drive, Virginia Beach, VA, USA; ^3^School of Medical Diagnostic and Translational Sciences, College of Health Sciences, Old Dominion University, Monarch Way, Norfolk, VA, USA

## Abstract

While induced pluripotent stem cells (iPSCs) hold great clinical promise, one hurdle that remains is the existence of a parental germ-layer memory in reprogrammed cells leading to preferential differentiation fates. While it is problematic for generating cells vastly different from the reprogrammed cells' origins, it could be advantageous for the reliable generation of germ-layer specific cell types for future therapeutic use. Here we use human osteoblast-derived iPSCs (hOB-iPSCs) to generate induced osteoprogenitors (iOPs). Osteoblasts were successfully reprogrammed and demonstrated by endogenous upregulation of Oct4, Sox2, Nanog, TRA-1-81, TRA-16-1, SSEA3, and confirmatory hPSC Scorecard Algorithmic Assessment. The hOB-iPSCs formed embryoid bodies with cells of ectoderm and mesoderm but have low capacity to form endodermal cells. Differentiation into osteoprogenitors occurred within only 2–6 days, with a population doubling rate of less than 24 hrs; however, hOB-iPSC derived osteoprogenitors were only able to form osteogenic and chondrogenic cells but not adipogenic cells. Consistent with this, hOB-iOPs were found to have higher methylation of PPAR*γ* but similar levels of methylation on the RUNX2 promoter. These data demonstrate that iPSCs can be generated from human osteoblasts, but variant methylation patterns affect their differentiation capacities. Therefore, epigenetic memory can be exploited for efficient generation of clinically relevant quantities of osteoprogenitor cells.

## 1. Introduction

Current therapies aim to replace autologous grafts with a biomimetic osteoinductive and osteoconductive scaffold coupled with an allogeneic or autologous osteogenic cellular component [1–3]. This would eliminate complications from donor site morbidity and promote faster healing and recovery times for patients. While choices for an osteoinductive and osteoconductive scaffold are plentiful, choosing a suitable osteogenic component proves to be more difficult. Mesenchymal stem cells (MSCs) are the current gold standard as an osteogenic component, as they have the capacity to differentiate into bone forming cells. Unfortunately, MSCs numbers shrink with the patient's age and environmental factors (e.g., smoking) and MSCs have a limited expansion capacity in vitro [4–6]. This poses significant challenges for the production of therapeutic quantities of cells from primary adult MSCs for osteogenic therapies [7–9]. With the discovery that using four transcription factors can successfully revert somatic cells back to a pluripotent embryonic state [10–12], many exciting therapeutic possibilities become available. iPSCs have the potential to overcome many of the shortcomings of current cell therapy strategies as they can proliferate infinitely in proper culture conditions making expansion to large quantities that are readily differentiated into cells such as MSCs and osteoprogenitors [13–16]. Another advantage of iPSCs is their ability to be used both in banks of HLA matched allogeneic cells [17–19] and autologously to generate therapeutic cells. Thus, iPSCs generated from donor cells using nonintegrative technologies (mRNA, Sendai, Episomal, etc.) could be a promising source of osteogenic cells [20–23]. However, an important topic still to be deciphered is variations seen during the iPSC reprogramming process that result in reduced, misdirected, or preferential differentiation capacity [15, 23–30]. Previous studies have found that a cause for some of these preferential differentiations is the remaining lineage-specific epigenetic (histone or DNA methylation mediated) profile of the iPSC, facilitating the cells differentiation capacity to favor the originating parental cell's germ-layer lineage [23, 28–32]. While previous studies have focused on the elimination of all residual epigenetic signatures, we propose here to take advantage of this parental cell memory as a means to preferentially enrich and expand a cell population of interest. Thus, reprogramming would provide an intermediate pluripotent master cell bank capable of limitless expansion of cells that are more readily differentiated back into their original cell type. The idea of a lineage-specific iPSC bank of cells for differentiation into the origin cell type, as compared to banks of multilineage capable iPSCs, would increase the likelihood of a successful differentiation and also reduce (but not eliminate) the burden of screening for fully reprogrammed cells [28, 30, 31].

Here we wished to determine if primary human osteoblasts are a valuable cell type for reprogramming and differentiation in comparison to adult human fibroblasts (hFB). While previous studies have examined the ability of various cell types including MSCs to be reprogrammed, it has yet to be determined if primary osteoblasts can be induced into a pluripotent state [14–16, 24, 27, 33]. As a source for the reprogramming process, osteoblasts provide a homogenous initial cell population as they are easily isolated from dense bone tissue from deceased donors after the loose bone marrow MSCs and hematopoietic cells are washed away. We further coupled the Taqman hPSC Scorecard analysis and the embryoid body (EB) differentiating technique to analyze the differentiation capacity of these iPSCs [22, 24, 34]. Using methylation analysis we discovered variations in methylated genes compared to other mesodermal origin iPSCs demonstrating an epigenetic pattern that could be used to identify mesodermal somatic memory effects. We further deciphered the capacity of these germ-layer memory affected cells to differentiate into osteoprogenitors without the ability to form adipocytes, for large-scale therapeutic productions. These data indicate that while these cells are epigenetically imprinted from their parental origins, their capacity to form osteogenic cells is unaffected.

## 2. Materials and Methods

### 2.1. Cell Culture

Human osteoblasts (hOB) were isolated from iliac crest bone tissue of 11 research-authorized donors from LifeNet Health (Virginia Beach, VA), The donor cells used for these experiments were consented and authorized for use in research purposes. All donors undergo rigorous screening of health history and infectious and genetic disorders prior to cell extraction. To extract cells, the bone was broken up and the marrow components were eliminated with washes and centrifugation. The remaining bone chips were then placed in growth media composed of DMEM with glutamine (Thermofisher), 10% FBS (Gibco), and 1% antibiotic-antimycotic (ABAM) (Sigma) to expand the osteoblasts. After isolation from the bone chips, hOBs were maintained in the media for 10–15 days without passaging (passage 0) before qRT-PCR analysis and passaging onto separate culture plates for calcium deposition assays. iPSCs were cultured on MEF (Globalstem) in KO-SR media (Thermofisher) or on Geltrex (Thermofisher) in mTeSR1 media (Stem Cell Technologies). hMSCs (Lonza) were cultured on CellStart CTS (Gibco) in StemPro MSC SFM Xenofree (Gibco) or standard growth media. Normal human osteoblasts (NHOst, Lonza) were grown in manufactures recommended media (Clonetics™ OGM™ Osteoblast Growth Medium, Lonza). For expansion experiments all cells were passed in log-phase of growth (~75–80% confluent), counted during passaging using a hemocytometer, and replated at 10 × 10^5^.

### 2.2. iPSC Reprogramming

hOBs (Passage 2) were reprogrammed as described in CytoTune-iPS Reprogramming Kit manual (Thermofisher). Briefly 2 × 10^5^ osteoblasts were infected with reprogramming cocktail at a multiplicity of infection (MOI) of 3 for 24 hours. After 7 days culture in culture media, the cells were passed onto MEFs and cultured in KOSR media for approximately 14–28 days until colonies emerged. Individual colonies were selected based on TRA-1-81 staining, expanded, and fully characterized before use. Reprogramming efficiencies were calculated by dividing successfully TRA-1-81 (Stemgent) staining colonies by the total number of cells plated onto final MEF feeder layers. Before use in experiments, iPSCs were passaged with Accutase (Stem Cell Technologies) onto Geltrex (Thermofisher) and maintained in mTeSR1 media for two passages.

### 2.3. Embryoid Body Differentiation

As previously described [13, 23, 24], hOB-iPSCs were exposed to dispase, at 37°C for 7 minutes, placed onto nonadherent dishes, and cultured for 7 days in EB media containing DMEM with Glutamax, 15% FBS, and 1% ABAM to allow EB aggregate formation. After 7 days in culture, EBs were seeded onto dishes with 0.1% gelatin and cultured for 7 subsequent days in EB media.

### 2.4. Osteoprogenitor Differentiation of iPSC

iPSCs were passaged using Accutase into single cell suspension and plated onto Geltrex coated plates in 20% Knock-out serum replacement media. Twenty-four hours following successful plating, the media was changed to DMEM/F12 with Glutamax supplemented with 20% FBS and 1% ABAM. Cells were allowed to grow with media replacement every third day for 7 days and passaged 1 : 4 until a uniform mesenchymal-like morphology was observed (3–5 passages). These cells were then subcultured in DMEM, 10% FBS, and 1% ABAM until use in further experiments.

### 2.5. Osteogenic, Adipogenic, and Chondrogenic Differentiation

OBs, iOPs and hMSCs were cultured with osteogenic differentiation media containing DMEM, 10% FBS, 1% ABAM, 50 *μ*M Ascorbate-2-Phosphate (Sigma), and 10 mM *β*-glycerophosphate (Sigma) for 21 days with media changes every 2 days. For adipose differentiation, the cells were cultured with adipogenic media containing DMEM, 10% FBS, 1 *μ*M dexamethasone, 1 *μ*M indomethacin, 200 *μ*M isobutyl-1-methylxanthine, and 10 *μ*g/mL insulin for 14 days with media changes every 2 days. For chondrogenic differentiation, the cells were passaged and counted, and 4 × 10^5^ cells were centrifuged at 300 ×g for 5 min in a 1 mL v-bottom tube. Resulting pellets were carefully resuspended in chondrogenic differentiation media (StemPro® Chondrogenesis Differentiation Kit, Life Technologies) and allowed to differentiate for 14 days with media changes every 2 days.

For Alizarin Red S (Sigma) staining, cultures were fixed with 10% formalin for 10 minutes and then washed with 1x PBS. The cells were then incubated with the Alizarin Red solution for 2 minutes and then rinsed with distilled water and covered in 1x PBS for imaging. For Oil Red O staining of lipids, differentiation wells were fixed with 10% formalin for 10 minutes, rinsed with PBS, and stained with Oil Red O solution (Sigma) for 10 minutes. The cells were then washed with distilled water and imaged. For chondrocyte HC staining, the fixed cell pellets were embedded in paraffin and sectioned. The sections were first cleared of paraffin using xylene and rehydrated in gradients of alcohol and then stained with Alcian blue (Sigma) in 3% acetic acid; the sections were then stained with Alcian blue and then dehydrated with 95% ethyl alcohol, 100% ethyl alcohol, and xylene. The slides were then mounted and imaged.

### 2.6. qRT-PCR and Scorecard Analysis

Trizol (Thermofisher) digestion was used to extract RNA from the desired cells according to manufacturer's instruction. The purified mRNA was transcribed to cDNA with the RNA-to-cDNA kit (Thermofisher). All qPCR was performed with Taq-man probes (Thermofisher, Sox 2; Hs01053049_s1, Oct4 (POU5F1); Hs00999634_gH, NANOG; Hs04260366_g1, hTERT; Hs00972656_m1, BMP7; Hs00233476_m1, Actin A2; Hs00426835_g1, PPAR*γ*; Hs01115513_m1, LEP; Hs00174877_m1, LPL; Hs00173425_m1, ADIPOQ; Hs00605917_m1, osteocalcin (BGLAP); Hs01587814_g1, Osteopontin (GZMB); Hs01554355_m1, RUNX2; Hs00231692_m1, BMP4; Hs03676628_s1, COMP; Hs00164359_m1, ACAN; Hs00153936_m1, COL10A1; Hs00166657_m1, Sendai Virus Detection; Mr04269876_mr, Mr04269878_mr, Mr04269879_mr, Mr04269880_mr, and Mr04269881_mr). To determine differences in iPSC pluripotency and EB differentiation capacity, we used the Thermofisher's Taqman hPSC Scorecard Panel array. The array derives an algorithmic comparison of input pluripotent and EB differentiated iPSCs to its reference set of data that is comprised of the methylation status of genes and their expression levels found in (differentiated/undifferentiated) 20 ESC and 12 iPSC. Statistical significance was analyzed with a Student *T*-test or a one-way ANOVA with TUKEY post hoc test where appropriate.

### 2.7. Flow Cytometry

The cells were detached from culture plate with Accutase (Thermofisher) and 0.5–1 × 10^6^ cells/100 *μ*L were suspended in flow cytometry buffer (PBS, 3% FBS, 1% sodium azide). The cells were incubated with fluorescently conjugated antibodies for 1 hr at room temperature. Antibodies against TRA-1-81 (0.25 *µ*g/10^6^ cells; StemGent), TRA-1-60 (0.25 *µ*g/10^6^ cells; StemGent), and SSEA4 (1 *μ*g/10^6^ cells; Santa Cruz Biotechnology) were used. The hMSCs and iOPs were probed with antibodies to CD29 (10 *μ*g/mL; CALTAG), CD14 (5 *µ*g/mL, Thermofisher), CD166 (5 *µ*g/mL, Thermofisher), CD105 (5 *µ*g/mL, Thermofisher), CD90 (5 *µ*g/mL, eBioscience), CD44 (0.15 *µ*g/10^6^ cells; Thermofisher), CD45 (10 *µ*g/10^6^ cells; R&D Systems), and CD31 (4 *μ*L/10^6^ cells; Thermofisher). Unconjugated primary antibodies for SSEA4 and CD34 were detected with an APC-conjugated secondary antibody goat (Gt), anti-Ms IgG (5 *µ*g/mL; Thermofisher). The cells were assayed with the BD Accuri C6 flow cytometer. Isotype control antibody groups and unstained cells were used to gate the positive cells and ensure that the fluorescent compensation was correct.

### 2.8. Immunofluorescence and Alkaline Phosphatase Staining

Live iPSCs were labeled with TRA-1-60 and TRA-1-81 antibodies (1 : 100; StemGent) in mTeSR1 media following manufacturer's instructions and imaged with Zeiss Axio Observer Z1 microscope (see flow cytometry methods for antibody information). Briefly, the mTeSR1 media were removed and replaced with fresh media containing the TRA-1-60 or TRA-1-81 antibody. The cells were incubated for 30 minutes in the dark and washed twice with PBS, and then the mTeSR1 media were replaced. For alkaline phosphatase staining, iPSC colonies were fixed with 10% formalin and labeled with alkaline phosphatase (Fast Red Violet) staining kit (Stemgent) following manufacturer's instruction. Briefly the colonies were washed with PBS and Tween 20, and then incubated with the alkaline phosphatase substrate staining solution for 15 minutes. The solution was then removed and the colonies were washed with PBS twice before imaging with a camera.

Cells were fixed with 10% formalin (Sigma) for 10 minutes and washed with PBS. For IF staining, the cells were labeled with primary antibodies: mouse anti-Sox2 (1 : 100; Cell Signaling), mouse anti-Oct4 (1 : 100; Cell Signaling), mouse anti-*β*-III tubulin (5 *µ*g/mL; Abcam), mouse anti-SMA (1 : 100; Abcam), and mouse anti-AFP (5 *µ*g/mL; Abcam). Secondary antibodies, Donkey anti-mouse IgG TRITC conjugated antibody (1 : 1000; Abcam) and goat anti-mouse IgG-FITC conjugated antibody (1 : 1000; Abcam), were used and DAPI stain (Thermofisher) was used to label the cell nuclei.

### 2.9. Genomic DNA Methylation Detection

Cells were collected and subjected to Trizol genomic DNA extraction following the supplied protocol (ThermoFisher Scientific). Subsequently, the collected genomic DNA was used to perform a methylation sensitive enzymatic digestion (EpiTect Methyl II DNA Restriction Kit) followed by PCR using primers specific to the promoter/gene regions of interest (SA Bioscience). Following PCR amplification the Ct values were input into the SA Bioscience calculation and quality control spreadsheet for analysis. Ct differences of more than 3 from internal control digestions were excluded from analysis.

## 3. Results

### 3.1. Osteoblast Characterization

**Figure 1:**
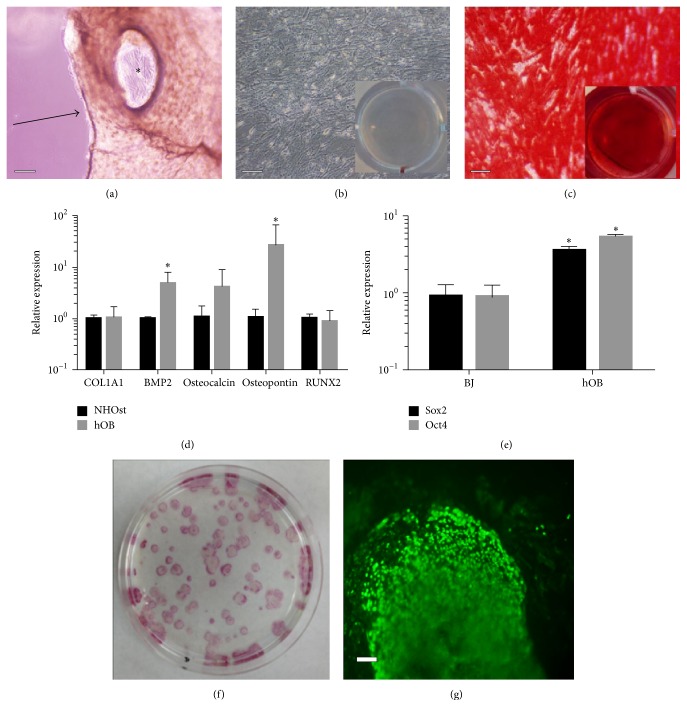
Characterization and reprogramming of donor-derived osteoblasts. (a) Example of donor bone chip in media (arrow) with osteoblasts emerging onto culture flask (asterisk). Osteoblasts were cultured in growth (b) or osteogenic induction media (c) and the resulting calcium deposits were stained with Alizarin Red S and imaged (scale bars = 30 *μ*m and inserts are at 1x). (d) The donor-derived osteoblasts were assayed with qRT-PCR for osteoblast markers, osteocalcin, osteopontin, RUNX2, BMP2, and COL1A1 and compared to a commercially available osteoblast cell line (NHOst). (e) qRT-PCR analysis of pluripotent gene expression, Sox2 and Oct4, in the hOBs compared to neonatal BJ fibroblasts. (^*∗*^*P* < 0.05). (f) Alkaline phosphatase staining of primary reprogramming plate of hOB-iPSC. (g) TRA-1-81 immunofluorescence of initial hOB-iPSC colony (scale bars = 30 *μ*m).

To extract primary human osteoblasts, bone chips from 11 donors were washed and processed thoroughly to remove all bone marrow constituents and then the chips were placed in culture dishes with growth media, which allowed for human osteoblasts (hOBs) to migrate out (Figure 1(a)). To confirm the identity of the donor-derived osteoblasts, cells were exposed to osteogenic media to induce calcium secretion and deposition. Alizarin Red S staining confirmed that cells grown in osteogenic media produced calcium deposition while cells cultured in nonosteogenic growth media did not (Figures 1(b) and 1(c)). To further confirm that our extraction process was isolating osteoblast populations, we examined the expression of a set of osteoblast specific genes. All 11 of our isolated hOBs showed significantly increased expression of osteopontin (*P* = 0.017) and BMP2 (*P* < 0.01) and similar levels of expression of RUNX2, collagen 1 A1, and osteocalcin as compared to the commercially available normal human osteoblast (NHOst) line (Figure 1(d)), indicating that our isolation procedure was successful.

### 3.2. Osteoblast Reprogramming

As many primary cell types are difficult to reprogram and some correlations have been established between initial pluripotency gene expression and reprogramming efficiency [24, 27], we examined the gene expression levels of Sox2 and Oct4 in our isolated hOBs. This revealed that the hOB cells had a significant increase of Sox2 and Oct4 gene expression as compared to the commonly reprogrammed BJ fibroblast cell line (Figure 1(e)), possibly indicating hOBs have a favorable reprogramming expression profile.

In order to reprogram hOB into an iPSC state, the cells were exposed to Sendai virus, in standard culture media, expressing the reprogramming factors (c-Myc, Sox2, Oct4, and Klf4). The cells were then cultured for 7 days before being passaged onto an inactivated mouse embryonic fibroblast (MEF) feeder layer. Successful reprogramming at the end of 4 weeks was determined based upon initial observation of a cellular conversion to a high nuclear to cytoplasmic ratio, colony formation with a well-defined border, and TRA-1-81 expression and subsequently by an initial alkaline phosphatase staining (Figures 1(f) and 1(g)). The total number of successfully reprogrammed initial colonies was counted prior to picking and the overall efficiency was calculated to be 0.1% for the hOBs, which was similar to the reprogramming efficiency obtained with fibroblasts at 0.13%. The selected hOB derived iPSCs (hOB-iPSCs) were further propagated on MEFs and frozen at various time points while being continuously screened during expansion for TRA-1-81 expression, indicating continued maintenance of pluripotency.

### 3.3. hOB-iPSC Characterization

**Figure 2:**
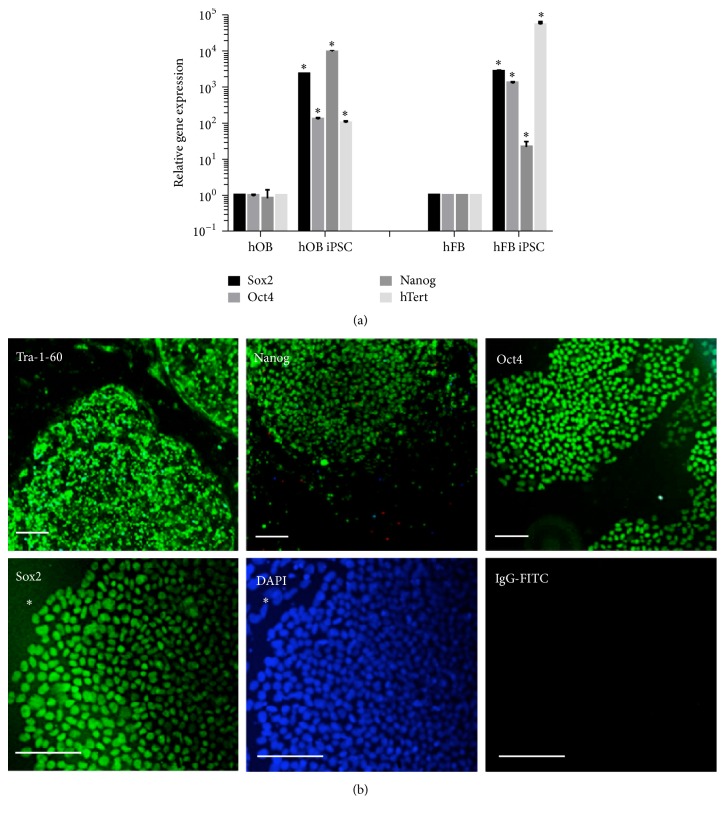
Pluripotent marker expression analysis of hOB-iPSCs and hFB-iPSCs. (a) hOB-iPSC and hFB-iPSC qRT-PCR expression of endogenous pluripotent genes, Sox2, Oct4, Nanog, and hTERT (^*∗*^*P* < 0.01). (b) Immunofluorescence of hOB-iPSC for pluripotency markers, TRA-1-60, Oct4, Nanog, Sox2, and IgG-FITC control (scale bars = 30 *μ*m). (*∗* indicates nonstaining MEF background.)

The hOB-iPSCs were then assayed for endogenous pluripotent gene expression of Sox2, Oct4, and Nanog (Figure 2(a)). The hOB-iPSCs had a significant increase in expression over the parental hOBs, with an approximate 1,000-fold increase in Sox2 expression (*P* < 0.001, ANOVA), 100-fold increase in Oct4 expression (*P* < 0.001, ANOVA), 10,000-fold increase in Nanog expression (*P* < 0.001, ANOVA), and 100-fold increase in hTERT expression (*P* < 0.001, ANOVA). To further confirm the protein expression the iPSCs were labeled with antibodies against Sox2, Oct4, Nanog, and TRA-1-60, which revealed high expression of all pluripotency associated markers (Figure 2(b)). To determine if there was residual Sendai virus gene expression in the reprogrammed cells, we performed a Sendai-gene specific qRT-PCR. As expected we saw no expression in all of our reprogrammed cell lines (data not shown).

### 3.4. hOB-iPSC Differentiation Capacity

To determine the differentiation propensity of the iPSCs for certain germ-layer lineages, the cells were analyzed with the Taqman hPSC Scorecard panel (Thermofisher). The Scorecard panel uses a robust algorithm based on the comparative methylation status, gene expression, and related differentiation propensity of 12 different iPSC lines, 20 ESC, and their differentiated progeny [34, 35]. The Scorecard panel initially examines the gene expression of undifferentiated and EB differentiated iPSCs for certain pluripotent (CXCL5, Nanog, Pouf51, Sox2, etc.), ectodermal (DRD4, PAX3, Sox1, Wnt1, etc.), mesodermal (HAND1, ESM1, BMP2, ODAM, etc.), and endodermal (FoxA2, GATA4, HNF1B, Sox17, etc.) markers. Then by inputting these values into an algorithmic assessment system, the expression data is assigned a score based on the comparisons to internal standards (established ESC and iPSC lines). Our analysis revealed that both hOB-iPSCs and hFB-iPSCs were pluripotent based on consistent algorithm scores. When differentiated into EBs, the EB hOB-iPSCs cells showed significantly (*P* < 0.05) increased algorithm scores associated with the ectodermal and mesodermal lineages, but not for endoderm (Table 1). This was in contrast to the hFB-iPSC EB cells, which scored positive for all three germ layers.

**Figure 3:**
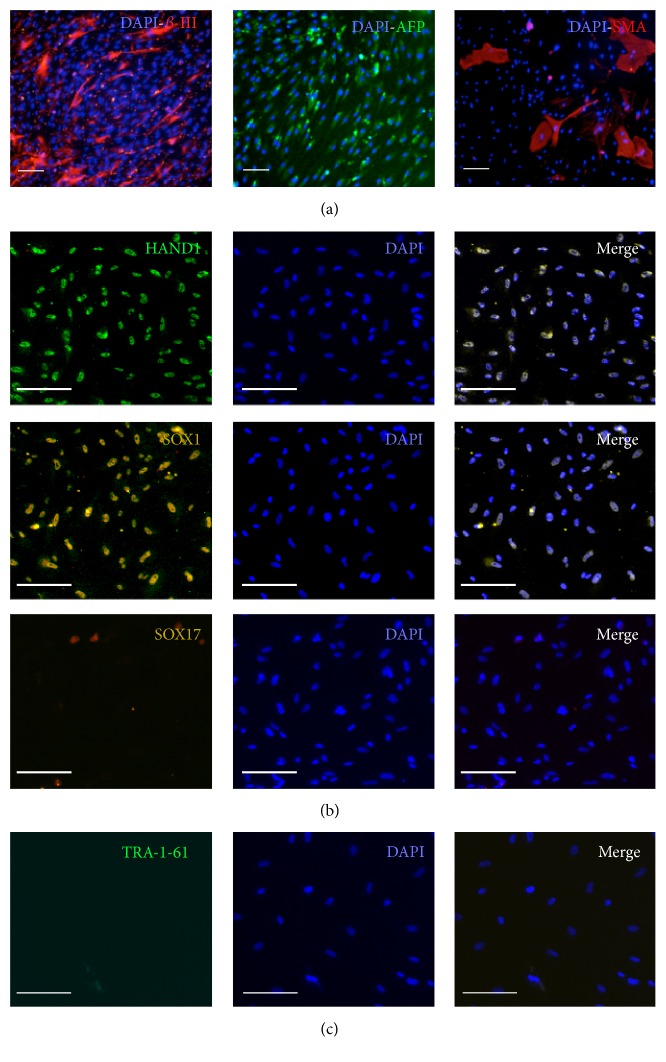
Differentiation of hOB-iPSCs into trilineages. (a) hOB-iPSC embryoid bodies (EBs) labeled with antibodies for cytoplasmic germ-layer markers, *β*-III tubulin (*β*-III; ectoderm), AFP (endoderm), and SMA (mesoderm). (b) hOB-iPSC EBs labeled with antibodies for germ-layer transcription factors, HAND1 (mesoderm), Sox1 (ectoderm), and Sox17 (endoderm). (c) Undetectable staining for pluripotent cells with TRA-1-60. Scale bars = 40 *μ*m.

Using immunocytochemistry we then confirmed the trilineage capacity of our hOB-iPSCs by examining the EBs expression of germ-layer associated cytoplasmic markers, *β*-III tubulin (ectoderm), Alpha Fetoprotein (AFP, endoderm), and smooth muscle actin (SMA, mesoderm) (Figure 3(a)). We successfully detected the presence of all three of these markers confirming these cells could make endodermal cells. However, we next stained for germ-layer specific transcription factors and identified strong staining for HAND1 (mesodermal) and Sox1 (ectodermal) but extremely low levels of the endodermal marker Sox17 (Figure 3(b)). Finally, to ensure we did not have any remaining pluripotent cells we also stained for the presence of TRA-1-61, which was undetectable (Figure 3(b)). These data aligned with our algorithm analysis indicate these cells lack complete differentiation capacity into the endodermal lineage.

### 3.5. Induced Osteoprogenitor Derivation

**Figure 4:**
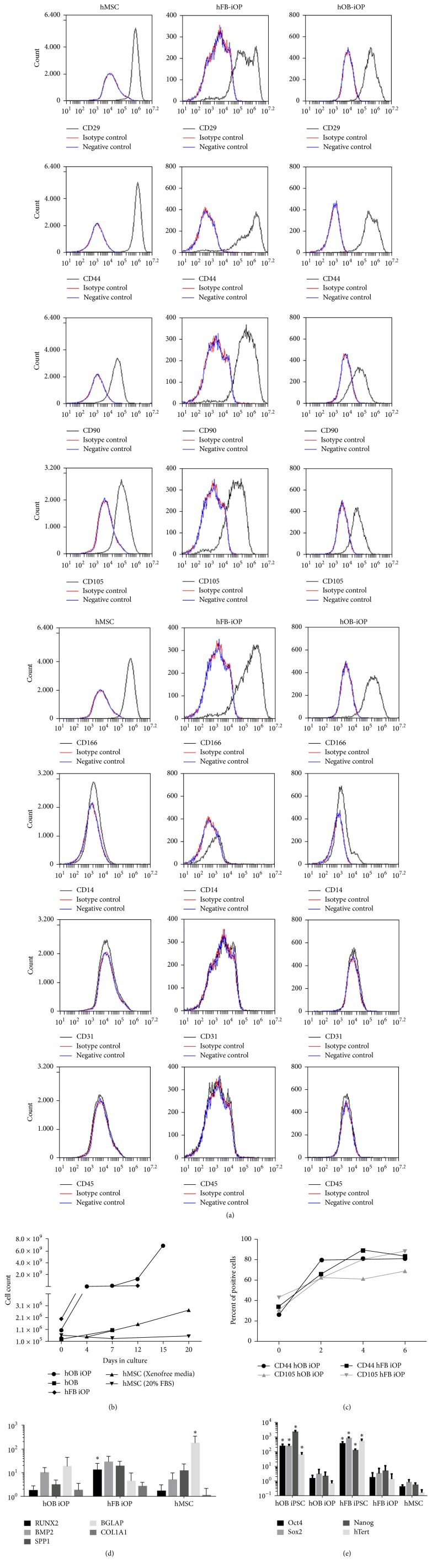
Induced osteoprogenitors (iOPs) cells differentiated from hOB-iPSC. (a) Flow cytometry analysis of iOPs and hMSCs revealed that the cells were positive for mesenchymal markers (CD29, CD44, CD90, CD105, and CD166) and negative for hematopoietic markers (CD14, CD31, and CD45) (black: CD marker expression, red: isotype control, and blue: unstained control). (b) Significantly (*P* < 0.05) faster growth kinetics of the hOB-iOPs and hFB-iOPs, as compared to hOBs and hMSCs in either 20% serum-containing media or Xenofree StemPro media at all time points. (c) Differentiation time of hOB-iPSCs to iOPs was assayed using flow cytometry for mesenchymal cell markers, CD44 and CD105. (d) iOPs were found to have similar levels of osteogenic related genes, RUNX2, BMP2, osteocalcin, and COL1A1 as compared to hMSCs *∗* indicates significant difference from the hOB-iOPs (*P* < 0.05). iOPs downregulated all pluripotency genes, Oct4, Sox2, Nanog, and hTERT indicating successful differentiations. *∗* indicates significant difference from the iPSC (*P* < 0.05).

In order to take advantage of the potential “parental cell memory” of these hOB-iPSCs, we further investigated the capacity of hOB-iPSC to form mesodermal origin osteoprogenitors (iOPs) cells. To accomplish this the hOB-iPSCs were differentiated using previously established differentiation protocols [36–38], using a single cell monolayer of iPSC cultured on 0.1% gelatin plates with serum-containing media, described here as induced osteoprogenitors (iOPs). Following two passages in progenitor growth media a consistent population of spindle-shaped mesenchymal cells emerged. Flow cytometry was conducted to determine if they exhibited the well-characterized cell surface protein markers indicative of cells of mesenchymal lineages (Figure 4(a)) [39–42]. The cells were positive for all mesenchymal-associated markers (CD90, CD105, CD166, CD44, and CD29) and negative for hematopoietic/endothelial cell markers, (CD14, CD45, and CD31) proving we had successfully generated iOPs.

As it is important for therapeutic purposes to be able to expand these cells to therapeutic quantities, we next examined the iOPs growth and propagation characteristics. Their doubling rate was found to be less than 24 hours, almost triple that of the hMSCs even when grown with higher FBS concentrations or in defined xenofree media. This high rate of growth would allow for these cells to be grown to over a 1000-fold increase in less than 10 days (Figure 4(b)), making them an easily scalable cell line.

To next determine the time necessary to see the emergence of a differentiated iOP population, the hOB-iPSCs and hFB-iPSCs were differentiated into iOPs and sampled at days 0, 2, 4, and 6 of the protocol. By day 2 of the differentiation, approximately 80% of the hOB-iPSC differentiating cells and 60% of the hFB-iPSC differentiating cells had begun to express the mesenchymal markers CD44, and 60% of both lines expressed CD105 (Figure 4(c)), suggesting they had differentiated into iOPs. The cells were then analyzed for gene expression of osteogenic associated markers (BMP2, osteocalcin, COL1A1, and RUNX2), which were found to be significantly upregulated in the iOP population with only BGLAP found to be significantly different as compared between the iOP and hMSC population (Figure 4(d)). As contaminating pluripotent cells pose a significant issue for deriving therapeutic cell populations we next assayed the cells for pluripotent markers Nanog, Sox2, Oct4, and hTERT. The iOPs showed a significant downregulation of all pluripotent genes back to levels comparable to the hMSCs indicating a lack of pluripotent cells in our differentiated populations.

**Figure 5:**
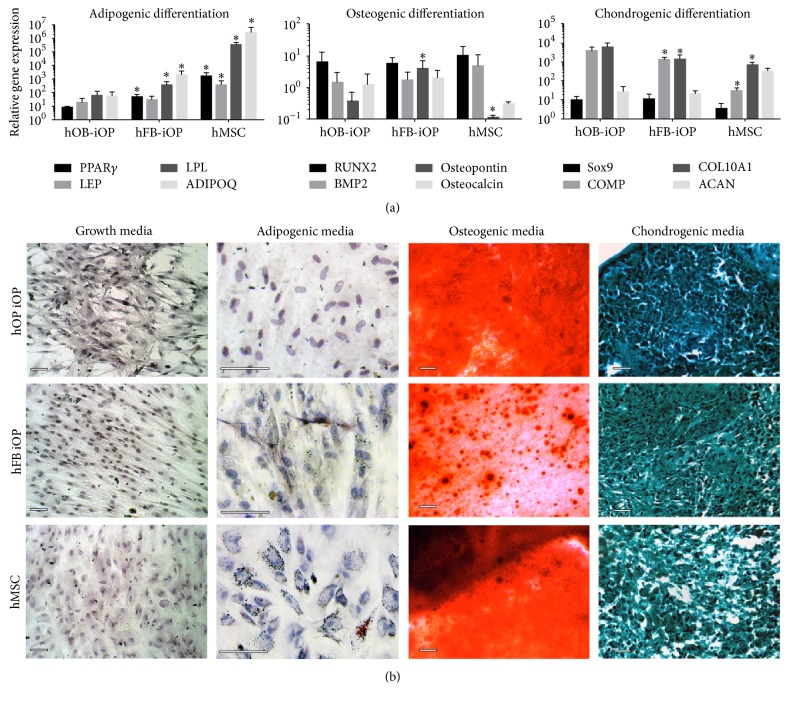
iOP differentiation to adipocytes, chondrocytes, and osteoblasts. (a) Gene expression of adipocyte, chondrocyte, and osteoblast markers normalized to their respective growth media control cells expression levels. Adipogenic expression levels were lower than both hFB-iOP and hMSC indicating a lack of adipogenic capacity  ^*∗*^Significantly different from hOB-iOP differentiated cells (*P* < 0.05). (b) Alizarin Red S, Oil Red O, and Alcian blue staining of adipogenic, osteogenic, and chondrogenic differentiations with a hematoxylin counterstain demonstrated comparable chondrogenic and osteogenic differentiation capacity, with a lack of hOB-iOP adipogenesis. Scale bars = 30 *μ*m.

As previous studies have noted that the generation of MSCs from iPSCs sometimes yields cells with only the capacity to generate chondrocytes and osteoblasts [38], we aimed here to explore if the germ-layer preferences we noted could possibly explain some of their findings. In order to examine the multipotency of the hOB-iOP population, we analyzed their osteogenic, chondrogenic, and adipogenic differentiation capacity. Following these differentiations the hMSCs and the hFB-iOPs had a significantly more adipocyte-related gene expression as compared to the hOB-iOP population (Figure 5(a)), while both the hMSCs and iOPs had similar levels of osteogenic and chondrogenic gene expression (Figure 5(b)). The differentiated iOPs and hMSCs were then stained for osteogenic calcium deposits with Alizarin Red S and adipogenic lipid droplets with Oil Red O and for chondrogenic glycosaminoglycans with Alcian blue (Figure 5(b)). This revealed that both hOB-iOPs and hFB-iOPs had similar amounts of Alizarin Red S staining compared to the hMSCs after 21 days (Figure 5(b)). The Oil Red O staining of the adipose differentiations showed the hMSC and hFB-iOPs with lipid vacuoles, while the hOB-iOPs showed undetectable staining (Figure 5(b)). The Alcian blue staining of our chondrogenic differentiations showed equal staining amongst the different cell groups. These data demonstrate that our hOB-iOPs could only differentiate into osteogenic and chondrogenic cell types, indicating that the original germ-layer deficiencies we found in these cells impacted their downstream capacity.

**Figure 6:**
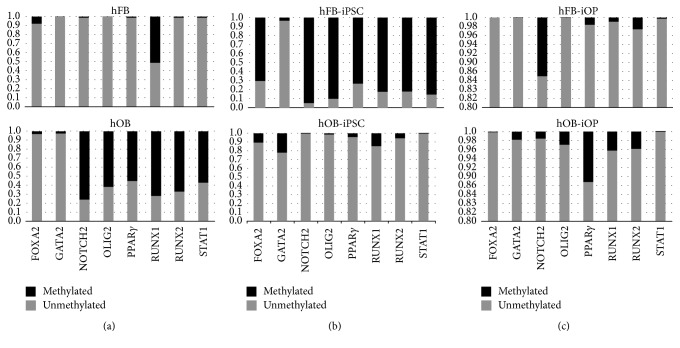
Promoter methylation analysis for unreprogrammed, parental iPSC, and iOP cells. Methylation sensitive enzymatic digestions were performed for hFB and hOB (a), hFB-iPSC and hOB-iPSC (b), and hFB-iOP and hOB-iOP (c). Graphs indicate promoter percent methylated (black) versus percent unmethylated (gray) of a subset of genes: endoderm (FOXA2, STAT1), ectoderm (OLIG2), and mesoderm (NOTCH2, GATA2, PPAR*γ*, RUNX1, and RUNX2).

We next wished to determine if our hOB-iPSC harbored some epigenetic variation that may be associated with their somatic origins that could be a causative factor in the preferential ectoderm/mesodermal differentiation preferences, which would also explain the lack of adipocyte differentiation capacity we were witnessing. To test this we preformed methylation sensitive qPCR for several gene promoters involved in fate determination for endoderm (FOXA2, STAT1), ectoderm (OLIG2), and mesoderm (NOTCH2, GATA2, PPAR*γ*, RUNX1, and RUNX2). Interestingly, we found a significant variation in the amount of methylation between the hFB and the hOBs at all stages of reprogramming and differentiation (Figure 6). Interestingly, NOTCH2, a key negative regulator of osteogenesis [43, 44], was significantly methylated in the hFB-iOPs as compared to the hOB-iOPs, while RUNX2 was methylated at similar levels at this stage (Figure 6(c)). Similarly, PPAR*γ*, a key regulator of adipogenesis [45], was significantly more methylated in hOB as compared to the hFB, both before reprogramming and after differentiation into iOPs (Figures 6(a) and 6(c)). Interestingly, the RUNX2 gene in hOBs had a higher percentage methylation than in the hFBs, contrary to what would originally be assumed (Figure 6(a)). These data together indicate the hOB-iOPs capacity to form both osteogenic and chondrogenic cells, but not adipocytes, which are likely due to the differential epigenetic regulation of these genes. The vast methylation differences between the hOB and hFBs and similar contrasts at the iPSC stage could also indicate a regulatory variation in the methylation on a more global scale during the reprogramming process.

## 4. Discussion

Previous iPSC reprogramming studies have used many different cell types including mesenchymal stem cells; however, to our knowledge, no other study has used osteoblasts to generate iPSCs. We show that osteoblasts are capable of being reprogrammed and do so at rates similar to BJ fibroblast cells, an unusual feature for a primary cell type, that typically are much more difficult to reprogram [24, 27]. One possible mechanism behind the hOB's reprogramming efficiency is the relatively elevated basal expression of the pluripotent master regulatory genes Oct4 and Sox2 in these cells. As previous studies have found that elevated expression of Oct4 can generate a primed state during reprogramming [46–48], here we demonstrated that the hOB-iPSCs were successfully reprogrammed as evident from both their morphological shifts coupled with endogenous expression of OCT4, SOX2, and Nanog and the cell surface markers TRA-1-81 and TRA-1-60. However, the differentiation capacity of these cells seemed to favor the mesodermal and ectodermal lineages. This finding indicated that the cells likely harbored some epigenetic memory that generated these preferential differentiation features as has been previously reported in the literature [23, 25, 26, 31]. Subsequently, when we analyzed the methylation status of several gene promoters important in fate specification, we found vast differences in the methylation patterning between the hFB and the hOB at all stages. These variations coupled with the algorithm scores, gene expression, and differentiation capacity indicate the cells are likely subject to some misappropriated epigenetic changes during the reprogramming process. Interestingly, we had noted while in active culture that the hOB-iPSCs had a tendency to spontaneously differentiate into mesenchymal-like cells, which had a very high rate of division. This supports the notion others have reported describing epigenetic variations that cause iPSCs to differentiate more easily into the same developmental lineage as the parental cells [23, 25, 26, 31]. While we showed that hOB-iPSCs differentiate as readily as hFB-iPSCs into iOPs, the cells lacked the ability to form adipocytes, similar to previous reports [38]. When we analyzed the methylation state of mesodermal fate control genes, we confirmed that the state of the adipose regulatory gene PPAR*γ* and the negative regulator of osteoblastogenesis NOTCH2 were methylated in hOB-iOPs, explaining the lack of adipogenesis. However, we unexpectedly found RUNX2 regulatory regions methylated at higher percentages than we were expecting in the parental hOB, possibly due to culture adapted, noncalcium producing state they are kept in during propagation. It has also been demonstrated that hyper- and hypomethylated regions within promoters have been shown to both negatively and positively regulate gene expression depending on the sites methylated. Thus, next step experiments should analyze site-specific methylation to determine if this is an issue. Furthermore, these patterns could indicate a general methylation difference on a more global scale impacting gene expression. These preferential differentiation tendencies of iPSCs are typically viewed negatively as the gold standard pluripotent cells, ESCs, lack epigenetic memory. However, lineage-specific epigenetic retention can be advantageous if it is able to generate cell types of parental origin without necessitating the addition of stimulating factors. By exploiting the tendency of hOB-iPSCs to revert back towards osteoprogenitor cells with high rates of proliferation, without the capacity to form adipocytes, large quantities of osteogenic cells can be produced using master banks of germ-layer specific iPSCs. As these iPSCs could be reprogrammed using the nonmutagenic, nonintegrative techniques demonstrated here, the induction genes would no longer be present following their successful generation, eliminating their possible contribution to future tumorgenesis. Further, the high rate of division we witnessed is similar to others who have noted that the default state of cells at any stage of development, but especially of those at a more primordial state, is to proliferate [49, 50]. Indeed, upon isolation into 2D culture, most nonproliferative cells will begin to divide without any cancer causing mutations being present [51] and without telomerase expression exhibiting a finite lifespan [9]. Thus, with careful screening the proliferation would be almost invariably viewed as a positive aspect of these cells, without any concern for unusually high rates of somatic mutations and thus tumorigenic cells. These cells can then be utilized alone, or with biological matrices and devices to further healing treatments. While choices for an osteoinductive and osteoconductive scaffold are plentiful, choosing a suitable osteogenic component proves to be more difficult. While MSCs, the current gold standard for bone cell therapies, have the capacity to differentiate into osteogenic cells, they also have the capacity to form adipocytes, unlike the osteoprogenitors presented here. Thus, having a more lineage-committed progenitor could lead to a quicker, more direct contribution to the regenerating bone with less off target differentiation. In future studies it will be important to determine if our findings are representative of a random reprogramming fault or if there is a specific recurrence of these markings within osteoblast populations, which could vary based upon the location they are isolated from. Furthermore, by expanding these studies to determine which cells have a predisposition to a certain lineage or cell type, incorporation of cells into biological and synthetic scaffolds may become simpler and require fewer manipulations to push the cells to the final cell type. Once these types of residual epigenetic marks have been vetted in in vivo applications as harmless, this strategy would ease the transition of these technologies into the clinic by simplifying differentiation procedures and the characterization of pluripotent cells. Regardless, if screening of cells is required, the methylation patterns here could suffice as a means to identify truly multipotent mesenchymal cells derived from iPSCs, adding to our bulk of knowledge about these variant germ-layer epigenetic profiles.

## 5. Conclusion

These studies for the first time reveal that human osteoblasts are capable of being reprogrammed back to a pluripotent state. Furthermore, these hOB-iPSCs harbored DNA methylation variants that resulted in restricted lineage capacity. While hOB-iPSC generated osteoprogenitors expanded rapidly, they were only capable of generating osteogenic and chondrogenic cells, lacking the ability to form adipocytes. If used therapeutically for cartilage or bone repair, this would eliminate the possibility of misdirected differentiation into fat. Together the data presented here demonstrates that the epigenetic memory effect found in reprogrammed hOBs could be used to advantageously generate an osteoprogenitor cell population with a more lineage-committed state for future therapeutic use.

## Figures and Tables

**Table 1:** Scorecard analysis of induced pluripotent stem cells.

	Passage	Pluripotent score	Ectodermal score	Mesodermal score	Endodermal score
hOB-iPSC	14	−0.03	−0.50	0.00	−0.42
hOB-EB	10	**−0.77**	*2.51*	*2.60*	0.46
hFB-iPSC	26	−0.56	−0.12	0.74	0.26
hFB-EB	20	**−2.14**	*6.10*	*3.62*	*1.32*

Bold: significantly downregulated; italic: significantly upregulated; normal font: not different from internal algorithm reference iPSC/ESC expression.
